# Thermal, Viscoelastic and Surface Properties of Oxidized Field’s Metal for Additive Microfabrication

**DOI:** 10.3390/ma14237392

**Published:** 2021-12-02

**Authors:** Rosendo Zamora, Juan Martínez-Pastor, Félix Faura

**Affiliations:** Departamento de Ingeniería Mecánica, Materiales y Fabricación, ETSII, Universidad Politécnica de Cartagena, E-30202 Cartagena, Spain; jm.pastor@upct.es (J.M.-P.); felix.faura@upct.es (F.F.)

**Keywords:** Field’s metal, oxide-coated liquid metals, surface properties, parallel-plate rheology, drop shape analysis, additive microfabrication

## Abstract

Field’s metal, a low-melting-point eutectic alloy composed of 51% In, 32.5 Bi% and 16.5% Sn by weight and with a melting temperature of 333 K, is widely used as liquid metal coolant in advanced nuclear reactors and in electro–magneto–hydrodynamic two-phase flow loops. However, its rheological and wetting properties in liquid state make this metal suitable for the formation of droplets and other structures for application in microfabrication. As with other low-melting-point metal alloys, in the presence of air, Field’s metal has an oxide film on its surface, which provides a degree of malleability and stability. In this paper, the viscoelastic properties of Field’s metal oxide skin were studied in a parallel-plate rheometer, while surface tension and solidification and contact angles were determined using drop shape analysis techniques.

## 1. Introduction

Low-melting-point metals, such as Hg, Ga, EGaIn and Galistan^®^, have proven to be very useful for certain applications [1]; for instance, in flexible electronics [2,3,4], electronic devices [5,6,7], fluidic microchannels [8,9,10], Cumby et al. [11] and Paracha et al. [12], in antennas, Ladd et al. [13], and in free-standing micro-structures, in the form of both wires and droplets. These liquid metals, except Hg, usually develop a very thin oxide film when they are exposed to an oxygen-rich atmosphere, which induces a deviation from liquid behaviour, as reported in previous works, such as Zamora et al. [14] and Dickey et al. [15], but allows these metals to form stable shapes [16]. This oxide skin, a few nanometres in thickness, was studied for Ga alloys by Larsen el al. [17] and for Indium alloys by Panek et al. [18] and Suzuki et al. [19]. It prevents the bulk material from further oxidation, but also affects the viscoelastic properties, surface tension and contact angle of this kind of metal. In the presence of oxygen and above its melting point, liquid Field’s metal also has an oxide skin and, as a consequence, its behaviour is dictated by the surface properties, conditioning its capacity to form droplets. For this reason, the rheological properties and the surface tension of Field’s metal were measured and analysed. In recent years, low-melting metal alloys have been used with other metals for new manufacturing applications. Allen and Swensen [20] tested a Field’s metal lattice encased in silicone rubber that could enable highly maneuverable robotic structures. Bismuth and indium alloys in particular, combined with other metals and materials, have been used instead of lead for metal solders due to their non-toxic properties. Indium-based alloys can also be used in applications where the alloy needs thermal conductivity. Due to its low melting point, wettability properties, ductility and fatigue resistance, indium alloys can be used in bending, anchoring or jig applications, but also to wet and weld both non-metallic and metallic surfaces.

In recent years, several authors have studied and characterized the behaviour of low-melting-point metals. Using oscillatory rheology with parallel-plates, Larsen et al. [17] studied the surface properties of EGaIn and concluded that the oxide skin has both elastic and viscous properties. Xu et al. [21,22] analysed the characteristics of this oxide layer with Ga and EGaIn, determining both their critical stress and surface energy in an acid bath with different concentrations of HCl, as well as in an inert gaseous atmosphere, to regulate the presence of an oxide skin and any effect on the viscoelastic behaviour. The literature contains several studies analysing properties such as the surface tension [23], and viscosity [24] of other liquid metals, such as alloys of Al, Sn, Zn, Bi and In. However, only the work of Lipchitz has dealt with Field’s metal [25], with the author concluding that further in-depth studies are needed on the dynamic properties of this material. Other essential aspects in the characterization of these liquid metals are the contact and solidification angles that the molten metals form on certain substrates, which is a key factor when assessing their possible technical applications [26]. In this respect, the studies of Liu et al. [27], Khan et al. [28], Kramer et al. [29] and Boley et al. [30] on variation in the contact angle of droplets on different substrates and oxidation conditions. However, the wettability properties of Bi and In alloys have not been studied as thoroughly, and only the works of Wang et al. [31] and Wu et al. [32] could be found. To date, no similar study has been developed for an oxidized Field’s metal. This work represents the first analysis of the surface properties of this low-melting-point metal alloy and its suitability for use in applications associated with droplet deposition.

## 2. Materials and Methods

A detailed experimental study was carried out on the properties of oxidized Field’s metal, to evaluate its influence on its overall behaviour. For this purpose, we first analysed Field’s metal dynamic properties by means of parallel-plate rotational rheology, including both oscillatory sweep and torsional flow, to determine the viscoelastic response of this alloy in the presence of the oxide skin. Secondly, its static properties were analysed, measuring its surface tension and solidification angle, using both sessile drop and pendant drop tensiometry. Similarly, the same measurements werecarried out in EGaIn (75.5% Ga and 24.5% In by weight), to compare the above-mentioned properties of both materials.

### 2.1. Surface Characterization and Thermal Parameters of Field’s Metal

As a preliminary study of Field’s metal, a chemical characterization of the oxide surface of Field’s metal was carried out using X-ray Photoelectron Spectrometry (XPS), performed using a Thermo-Scientific “K-Alpha” equipment, and the obtained data were analysed with Avantage Data System software to determine the composition of the oxide layer. Differential Scanning Calorimetry (DSC) was used to obtain its thermal parameters [32,33] using a Wettler-Toledo DSC822e DSC at a heating and cooling rate of 10 K/min over a temperature range from 298 to 348 K.

All XPS spectra were collected using Al-K radiation (1486.6 eV), monochromatized by a twin crystal monochromator, yielding a focused X-ray spot (elliptical in shape, with a major axis length of 400 μm) at 3 mA × 12 kV. The alpha hemispherical analyser was operated in the constant energy mode with survey scan pass energies of 200 eV to measure the whole energy band and 50 eV in a narrow scan to selectively measure the particular elements. XPS data were analysed with Avantage software. A smart background function was used to approximate the experimental backgrounds, and surface elemental composition was calculated from background-subtracted peak areas. Charge compensation was achieved with the flood gun system, which provides low-energy electrons and low-energy argon ions from a single source.

### 2.2. Rheological Characterization

The literature mentions a variety of rheological methods and types of rheometer [34] used to analyse the viscoelastic properties of liquid metals [24,35]. In this is work, a rotational rheology was used, following the same line of study as Dickey et al. [15,17] and Xu et al. [21,22], using a TA Instruments “AR-G2” rotational rheometer with a parallel-plate geometry of 25 mm diameter (both upper and lower plates were made of anodized aluminium). The gap size was varied from 1.4 to 2.4 mm for Field’s metal, and from 1.2 to 2.1 mm for EGaIn, depending on the amount of sample. All measurements of the viscoelastic properties of Field’s metal were performed at 348 K, and at 303 K in the case of EGaIn, with a common equilibration time of 30 s for all the samples. To study the linear response, oscillatory amplitude sweeps were made, while three different modes of the torsional flow test were followed to study the non-linear response (peak hold, steady flow and flow ramp). No pre-shear was applied in the rheological tests to minimize the shear history, and samples were loaded into the rheometer by syringe. The test parameters employed for each type of rotational technique are included in Table 1.

In addition, a flow temperature ramp was followed for both Field’s metal and EGaIn, imposing a constant shear rate of $5\times 10^{-3}$ s${}^{-1}$ (to ensure the stability of the torsional flow) and a thermal ramp rate of 1 K/min, within a temperature range of from 343 to 473 K for Field’s metal, and from 303 to 383 K for EGaIn.

### 2.3. Axisymmetric Drop Shape Analysis

Drop shape analysis involves a group of techniques, based on a numerical analysis of the shape and dimensions of a drop, to determine surface and interfacial properties, such as interfacial tension, surface energy or contact angle [36]. In the present study, pendant drop and sessile drop tensiometry were used to determine surface tension [21,22], and solidification or contact angles [28,29,30], respectively, since both are considered reliable methods. However, we also took into account the condition of a low-Bond number for the physical problem associated to this type of droplet, where gravitational forces can be negligible compared with interfacial forces [37,38,39]. For this drop shape analysis, we used a Krüss “DSA100” analyser, which has been used in similar works [40], and considered that the droplets preserve vertical symmetry (Axisymmetric Drop Shape Analysis; ADSA [41,42,43]). The experiments were performed using syringes with a 25 gauge needle, i.e., inner diameter of 0.26 mm and outer diameter of 0.515 mm, to generate sessile and pendant drops.

The ADSA for sessile drops was performed by generating droplets of Field’s metal (ρ = 7880 kg m${}^{-3}$) at 358 K, falling on a substrate at 358 K. After deposition of the droplet, the system was cooled from 358 to 318 K to obtain complete solidification of the sessile drop. Contact angles were measured using the ADSA software (version 1.90.0.14) of the DSA100 (Krüss) analyser [44,45] every 5 K.

The solidification and contact angles were determined by individually placing the droplets on a substrate in the presence of air (O_2_) and inert gas (N_2_), by means of a vertical syringe. The substrate temperature was corrected by measuring its temperature in direct contact with a thermocouple.

For Field’s metal, the syringe was heated to 358 K and sessile droplets were placed on substrates, which were also heated to reach 358 K. The substrates were of differing natures to represent insulating (glass) and conductive (AISI 316L steel) materials, and polymers (PTFE and resin). Note that the resin used in these experiments was a high-temperature photopolymer resin commonly used in stereolithography, with a heat deflection temperature (HDT) of 511 K at 0.45 MPa.

**Figure 1:**
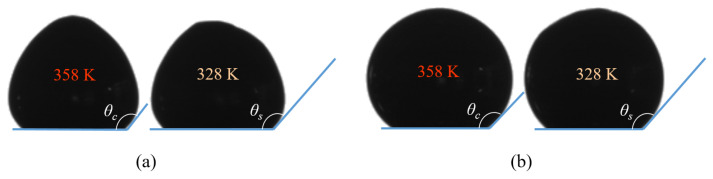
Examples of Field’s metal sessile drop images used in ADSA to determine solidification ($\theta_{s}$) and contact ($\theta_{c}$) angles: (**a**) O_2_. (**b**) N_2_.

After heating, the system was cooled from 358 to 318 K, and images were taken every 5 K interval (Figure 1). The same substrates were used for the EGaIn, but the experiments were performed at a constant syringe and substrate temperature of 298 K, so only the contact angle was measured.

Pendant drop tensiometry was used to obtain the surface tension value of Field’s metal, with and without an oxide skin, generating the droplets at 358 K in the presence of air or nitrogen, respectively. Pendant drops of different sizes were considered and measured by the ADSA software, due to the influence of the oxide skin on the surface tension values, as reported in previous works [21,22].

## 3. Results

### 3.1. XPS and DSC Measurements of Field’s Metal

**Figure 2:**
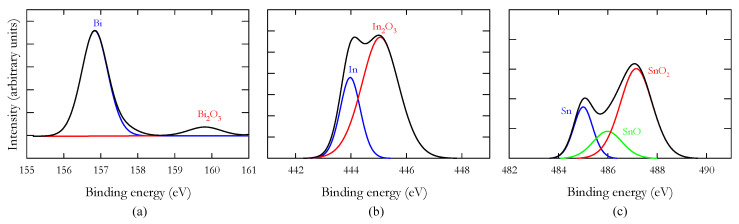
XPS binding energy results with the oxide assignments according to the referenced values included in Table 2 for: (**a**) Bismuth, (**b**) Indium and (**c**) Tin.

To study Field’s metal oxidation, a sample was melted and later solidified in a controlled atmosphere and oxygen content (2500 ppm). The presence of an oxide skin was clearly noticeable, even at low oxygen concentrations. The results obtained from the XPS analysis are summarised in Figure 2. The energy peaks can be assigned to Bi_2_O_3_, In_2_O_3_, SnO and SnO_2_ according to the references shown in Table 2.

The results obtained from the thermal analysis of the alloy are shown in Table 3.

### 3.2. Rheological Characterization of Field’s Metal

The rheology of liquid metals with an oxide skin is usually dominated by the surface stress of the film, which presents elastic properties and yield stress, with the bulk of the material usually being a Newtonian fluid of very low viscosity [35]. While the viscoelasticity of this oxide layer has been studied in detail for certain low-meting point metals, such as Ga or EGaIn, there are no previous studies on the response of this oxide layer in Field’s metal.

**Figure 3:**
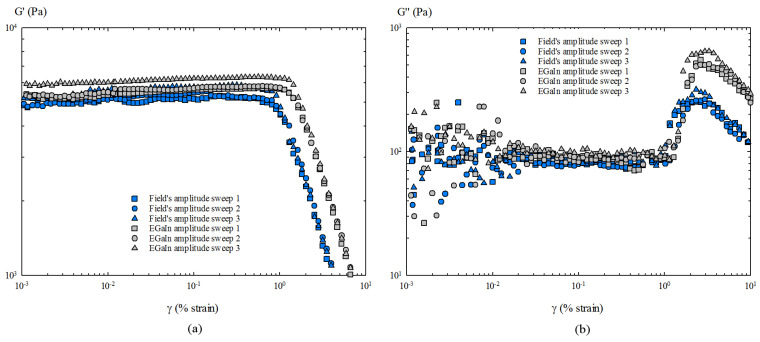
Results of the oscillatory amplitude sweep tests: (**a**) Storage modulus $G^{\prime }$. (**b**) Loss modulus $G^{\prime \prime }$.

Starting with a linear rheological analysis, the results of oscillatory amplitude sweeps are presented first (Figure 3). The noise appearing for $G^{\prime \prime }$, in the first decade of the amplitude strains, can be attributed to the accuracy of the rheometer at very low strain values and the loading procedure of the samples. As the tested samples did not occupy the entire surface of the 25 mm rheological plates, an effective test diameter of 24 mm was taken when transforming the oscillatory moduli to surface moduli [17,21].

In this respect, the linear viscoelastic region (LVR) of Field’s metal is defined in the range of $5\times 10^{-3}$ and 0.9% strain, with constant oscillatory modulus values of $G^{\prime }$ = 5358 ± 147 Pa and $G^{\prime \prime }$ = 84 ± 6 Pa, providing a phase angle of δ = 0.9° at 1 Hz.

The resulting values of the surface moduli are $G_{S}^{\prime }$ = 16.07 ± 0.44 Nm${}^{-1}$ and $G_{S}^{\prime \prime }$ = 0.25 ± 0.02 Nm${}^{-1}$. Similarly, for EGaIn, the LVR is defined between $6\times 10^{-3}$ and 1.1% strain, where $G^{\prime }$ = 5548 ± 141 Pa and $G^{\prime \prime }$ = 98 ± 14 Pa (δ = 1.01° at 1 Hz), and, consequently, $G_{S}^{\prime }$ = 16.64 ± 0.42 Nm${}^{-1}$ and $G_{S}^{\prime \prime }$ = 0.29 ± 0.04 Nm${}^{-1}$, in accordance with the results of previous works; i.e., $G_{S}^{\prime }$ = 13.3 ± 2.6 Nm${}^{-1}$ [17].

These results show that Field’s metal has a lower phase angle value, i.e, it is less viscous than EGaIn in the linear regime, even though it has a higher $G_{S}^{\prime }$ and, therefore, greater surface elasticity than EGaIn. For both liquid metals $G^{\prime }>>G^{\prime \prime }$ (and $G_{S}^{\prime }>>G_{S}^{\prime \prime }$) in the LVR, which shows that the elasticity of the oxide skin dominates the linear viscoelastic behaviour of this type of metals.

**Figure 4:**
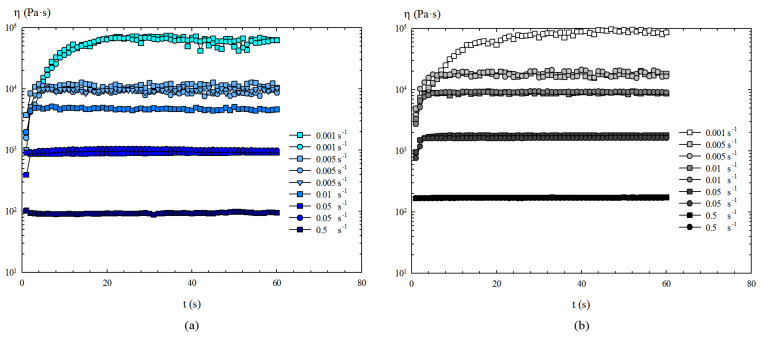
Shear viscosity obtained in torsional flow with peak hold: (**a**) Field’s metal. (**b**) EGaIn.

With regard to the non-linear rheological behaviour of these liquid metals, the results of the torsional flow with a peak hold of shear rate are presented first. Figure 4 shows how, by increasing the shear rate imposed during each test, not only are lower values of viscosity obtained, but also a decrease in the time required to reach steady state in the non-linear regime.

**Figure 5:**
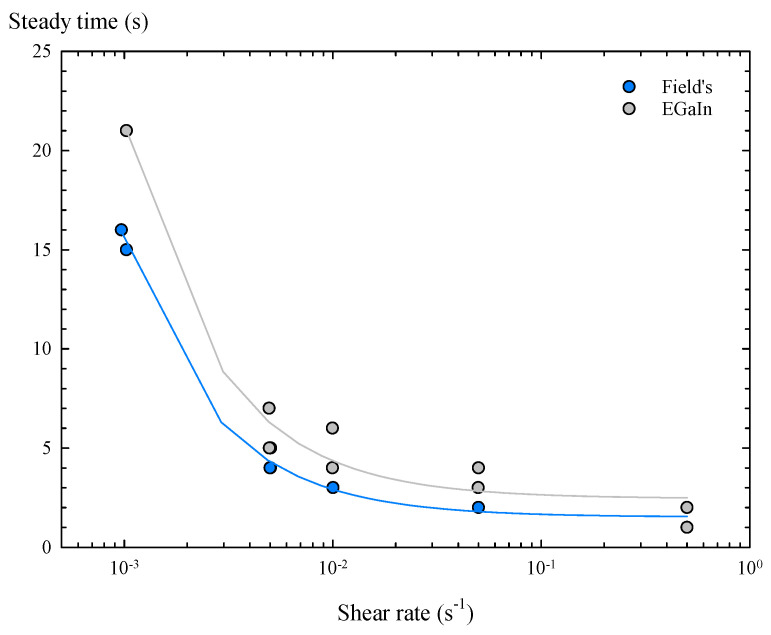
Time to reach steady state in torsional flow as function of shear rate: (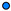) Field’s metal. (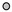) EGaIn.

Therefore, by representing the resulting steady times versus shear rate, a decreasing exponential trend is obtained for both metals (Figure 5), providing that, for shear rates above 0.5 s${}^{-1}$, the steady time is lower than 2 s for Field’s metal and lower than 3 s for the EGaIn. That is, in general, the oxidized Field’s metal reaches the steady state slightly faster than the oxidized EGaIn under shear flow.

Regarding the steady shear stress developed during these torsional flow tests, the maximum obtained value was 60.4 Pa for Field’s metal and 92.9 Pa for EGaIn. This is also indicative of the fact that Field’s metal develops lower shear stress levels for a given shear rate than EGaIn, meaning that Field’s metal has lower viscosity than EGaIn in liquid state in the presence of the oxide skin. This agrees with the values of the phase angle provided by the oscillatory amplitude sweeps.

**Figure 6:**
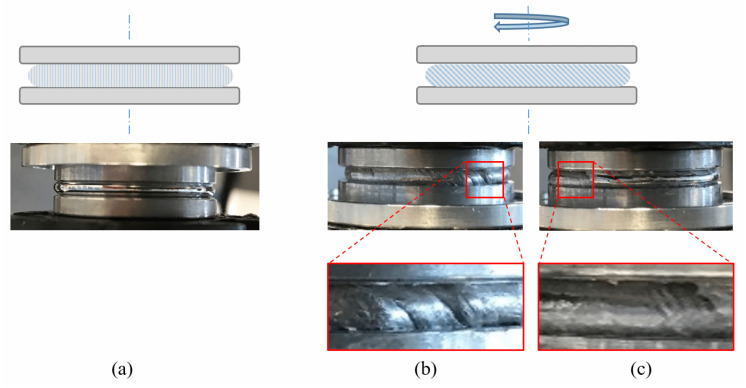
Graphic scheme and pictures of liquid metal samples between the two parallel plates of the rotational rheometer during torsional flow tests: (**a**) Initial state. (**b**) Detail of buckling of the oxide skin of Field’s metal and (**c**) EGaIn.

During the torsional flow tests, stretching phenomena were observed at the oxide skin of the rheological samples (Figure 6). As previously reported by Xu et al. [21] for EGaIn, buckling lines were evident in the oxidized Field’s metal, which indicates partial or apparent cracking of the oxide skin when yielding. However, the presence of oxygen in the surrounding atmosphere is able to renew the oxide skin, maintaining such metals’ capacity to keep flowing.

**Figure 7:**
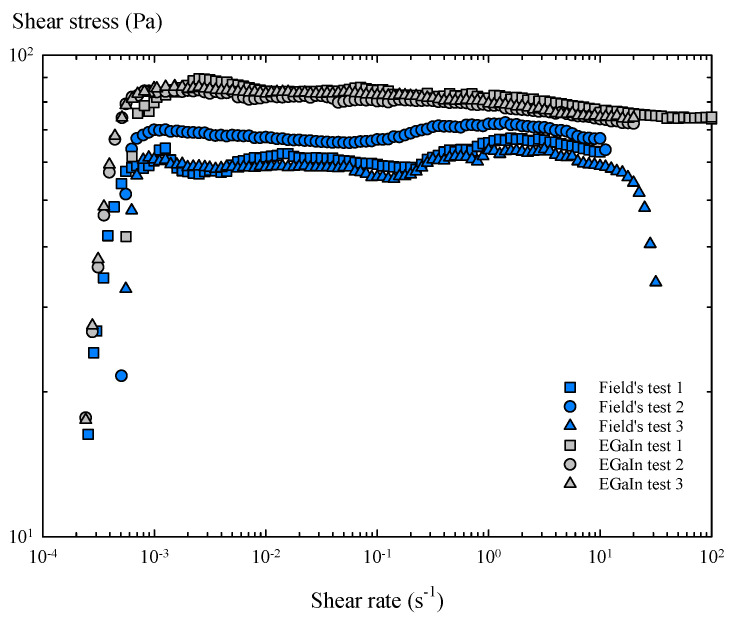
Flow curves of Field’s metal and EGaIn obtained in steady torsional flow tests.

In the steady torsional flow tests, the rotational rheometer applies a ramp of shear rates, until the samples reach a steady state. Considering the results obtained in torsional flow with peak hold, a characteristic sampling time of 5 s was imposed for this group of tests. These experiments (Figure 7) confirmed that the oxide skin provides Field’s metal with a critical shear stress, which is virtually constant over a wide range of shear rates, as seen in similar studies [21,22].

**Figure 8:**
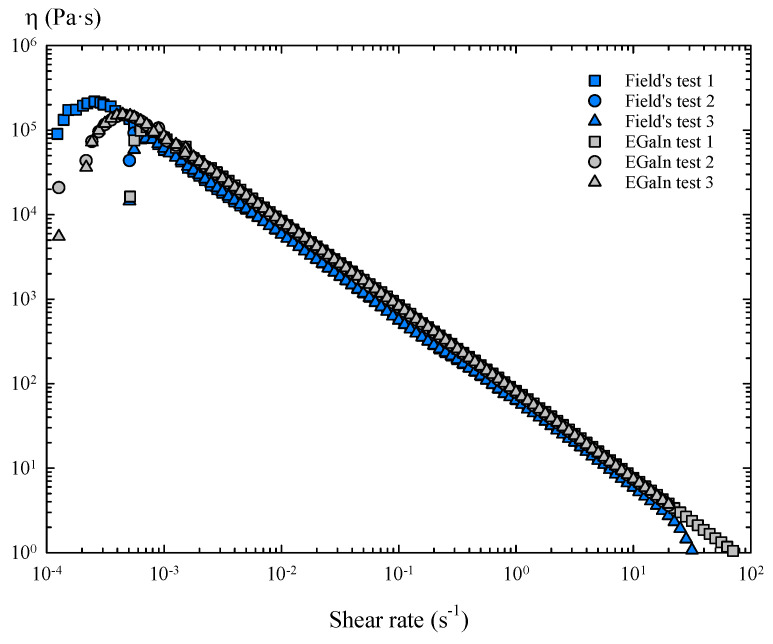
Viscosity curves of oxidized Field’s metal and EGaIn obtained in steady torsional flow tests.

Therefore, in the presence of air, the oxide skin of these metals generates a characteristic and constant tension during shear flow, i.e., yield stress. As a consequence, these oxidized layers show solid-like behaviour below the yield stress, but a perfectly plastic response above it, as the shear stress stops increasing with the shear rate, keeping the stress value practically constant. The resultant shear viscosity of the oxidized Field’s metal shows a shear thinning behaviour and slightly lower viscosity values than the oxidized EGaIn over a wide range of shear rates (Figure 8).

**Figure 9:**
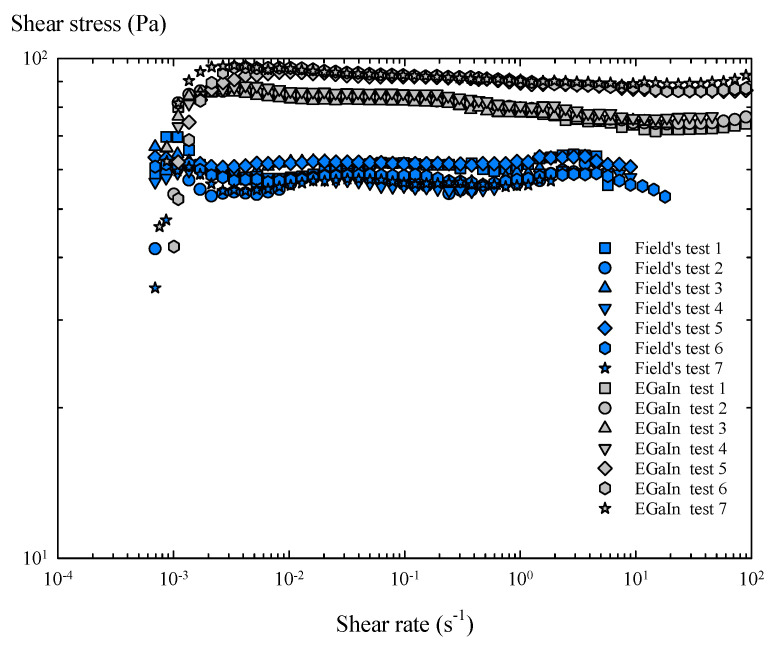
Flow curves of Field’s metal and EGaIn obtained in the torsional flow ramp tests.

**Figure 10:**
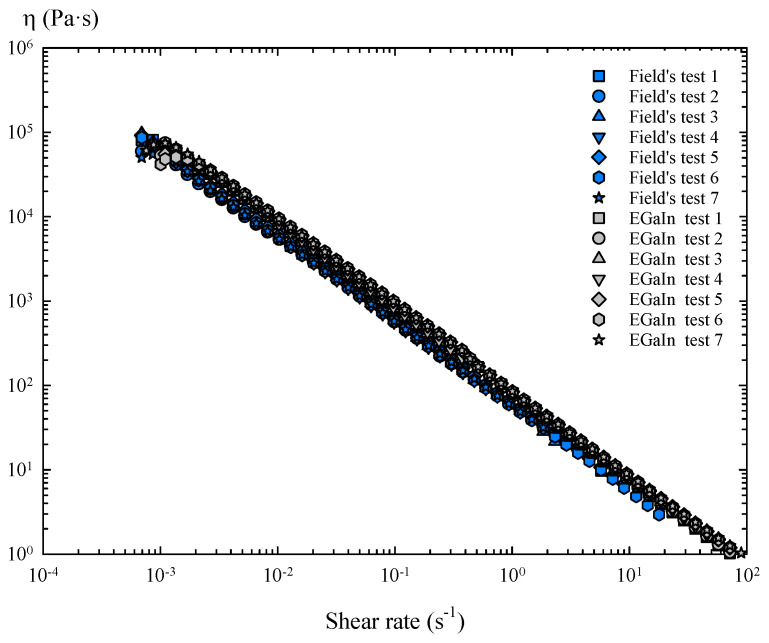
Viscosity curves of oxidized Field’s metal and EGaIn obtained in the torsional flow ramp tests.

For the rotational tests with torsional flow ramp (Figure 9 and Figure 10), the results were very similar to those obtained with a steady torsional flow, and the oxide skin also maintained the shear stress value after a certain critical shear rate was exceeded during the flow ramp. However, the yield stress values achieved in each flow test vary slightly (Figure 7 and Figure 9), which can mainly be attributed to the differences between samples due to their manual loading in the rheometer.

The shear viscosities of these oxidized metals follow the same behaviour as the steady torsional flow tests (Figure 8 and Figure 10).

**Figure 11:**
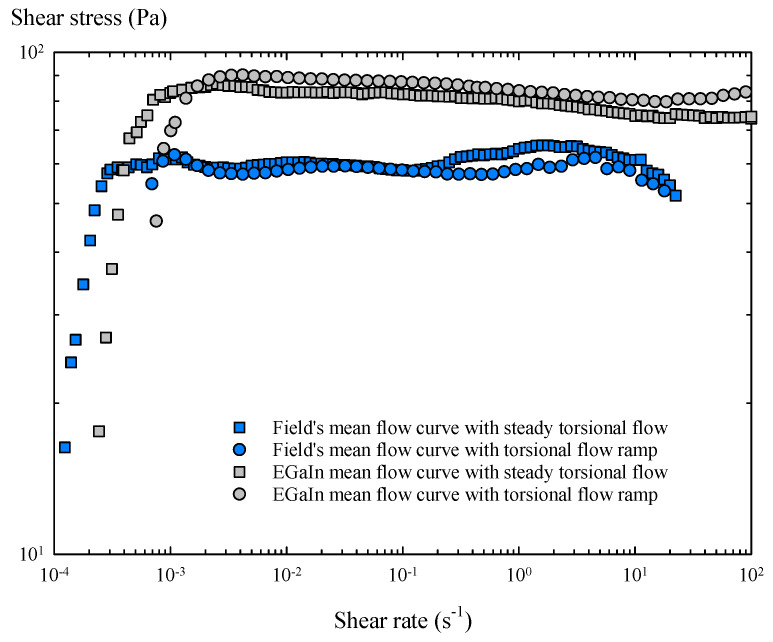
Averaged flow curves of Field’s metal and EGaIn obtained in steady torsional flow and torsional flow ramp tests.

Finally, the results obtained with a steady torsional flow and a torsional flow ramp were compared, considering the mean flow curve obtained with all the samples of each metal (Figure 11).

Several useful results can be extracted from the averaged flow curves. The oxidized Field’s metal is less viscous in molten state than the oxidized EGaIn, and starts to flow at lower shear rates and develop a lower value of yield stress. In contrast, Field’s metal is slightly less stable during shear flow, as shear stress tends to fluctuate with increasing shear rate. On the other hand, EGaIn shows a less variable flow curve, with less pronounced fluctuations and a smooth increase in shear stress before yielding, indicating that the oxide layer of EGaIn is slightly more stable in shear flows.

The mean yield stress values of both metals were determined, taking the mean value of all the shear stress data obtained in the averaged flow curves above the critical shear rate. For Field’s metal, the mean yield stress was 55 Pa in steady torsional flow, and 58.7 Pa with the torsional flow ramp, providing a nominal value of 57 ± 5 Pa. Similarly, for EGaIn, the mean yield stress in steady torsional flow was 85.5 Pa and 89.1 Pa with the torsional flow ramp, providing a nominal value of 87 ± 5 Pa. The main results of this rheological study are summarized in Table 4, where they are compared with the corresponding values taken from previous works.

**Figure 12:**
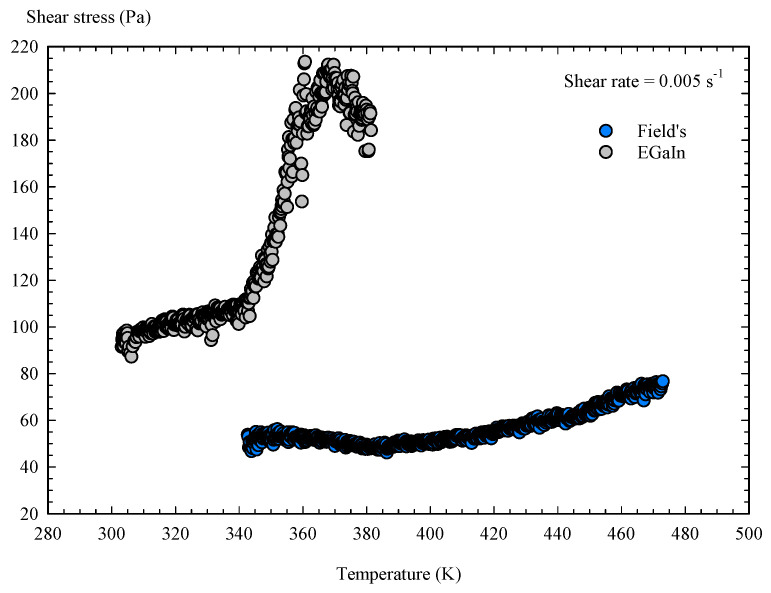
Results of the flow temperature ramp tests.

Finally, a temperature flow ramp was applied to both liquid metals with oxide skins (Figure 12) to analyse the influence of temperature on shear stress and viscosity. In the temperature sweep from 343 to 473 K, in the presence of an air atmosphere, Field’s metal maintained its shear stress at a stable level of approximately 55 ± 10 Pa from its melting point to 438 K. Above 438 K, the oxide layer gradually degrades, becoming scorched and unable to sustain further renewal, while also changing its colour and appearance. In the case of EGaIn, applying a temperature sweep from 303 to 383 K, the shear stress followed a stable trend up to 341 ± 2 K, above which the oxide skin sharply degraded, leading to an abrupt increase in the shear stress and a rapid loss of its rheological surface properties. Once degraded, the appearance of the oxide skin of both metals was quite similar. For Field’s metal, we found a minimum value of shear stress at 382 ± 2 K, which can be taken as the optimal processing point for this material when yielding in liquid state (minimum shear viscosity). In contrast, the shear stress and viscosity of EGaIn always showed an increasing trend as the temperature increased from its melting to 341 K, which constitutes the critical point for using this liquid metal.

### 3.3. Axisymmetric Drop Shape Analysis

This section describes how ADSA of sessile drops was used to determine the solidification and contact angles, and the effect of the overheating, since these parameters define the wettability of these low-melting-point metals [45,53].

In the presence of an oxygen-rich atmosphere (O_2_), the presence of an oxide skin meant that the droplets of Field’s metal did not take on a spherical form (Figure 1a), as reported previously by Hutter et al. [8] and Khan et al. [28] using EGaIn. By contrast, in the presence of a nitrogen-rich atmosphere (N_2_), the non-oxidized droplets of Field’s metal tended to generate spherical shapes, with higher values of solidification angles (Figure 1b). Certain contractions and shape changes were detected on the upper part of the droplets, once the temperature fell below the melting point (see 328 K photographs on the right of Figure 1a,b). No further variation in the contact angle was detected below 328 K.

To compare the liquid metals, the following table summarizes the experimental values of the solidification and contact angles obtained on the cited substrates (Table 5). The results demonstrate that the oxide skin increases the wettability of both metals in liquid state, leading the drops to have lower contact angles, as reported previously [27,28,29]. In the presence of N_2_, both metals showed similar behaviours, and higher contact angles are reached with all the substrates used.

It can be observed that when the oxide skin is present, the contact and solidification angles generally increases along with surface roughness (see $R_{a}$ in Table 5). The results show that the contact angle was strongly affected by the oxide skin when glass and steel substrates were used (decreasing by around 7 ± 1°). On the other hand, when polymeric substrates were used (PTFE and resin), the contact angle only decreased by around 1.5 ± 0.5°.

**Figure 13:**
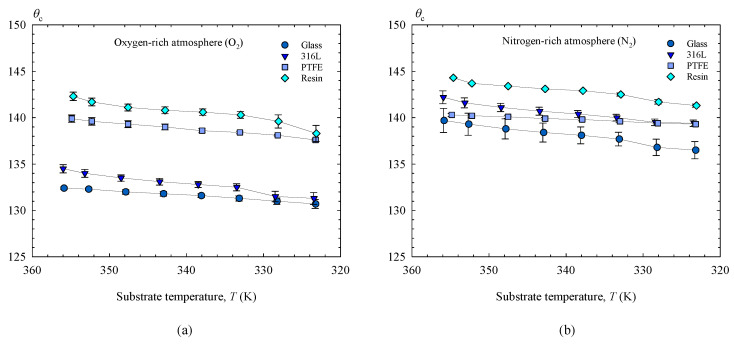
Contact angles ($\theta_{c}$) of Field’s metal as function of substrate temperature, for different types of substrate (AISI 316L, Resin, PTFE, Glass): (**a**) O_2_. (**b**) N_2_.

The contact angles of Field’s metal are represented in Figure 13 as a function of substrate temperature in order to evaluate the effects of overheating and solidification on its wettability. Similar variations were measured in both cases reaching a maximum decrease of 4° in a range of 32 K. The solidification angles obtained for each substrate are included in Table 5.

**Figure 14:**
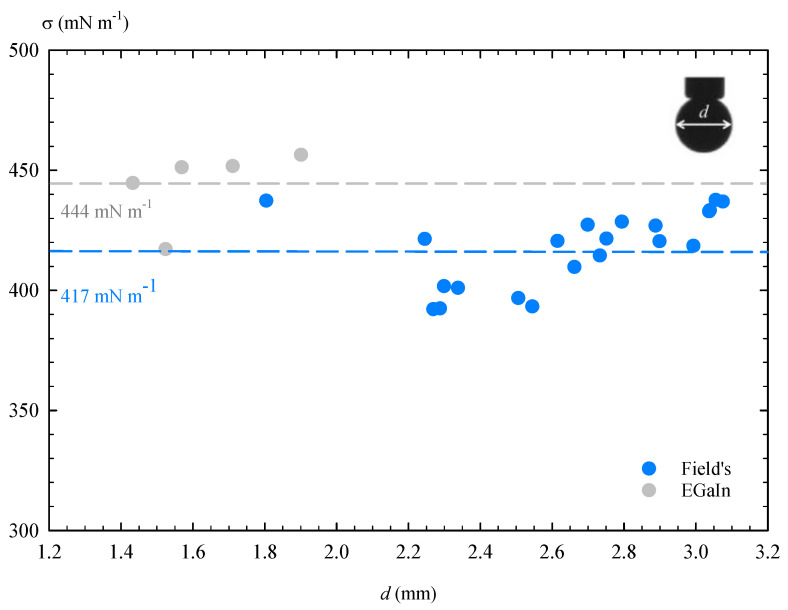
Surface tension (σ) of Field’s metal and EGaIn in inert N_2_ atmosphere.

Pendant drop tensiometry was used in order to determine the interfacial tension of Field’s metal [39,40]. In the presence of N_2_, the oxide skin of these liquid metals is not formed and the surface tension of the bulk of the material can be determined by ADSA. In these experiments (Figure 14), a mean value of 417 mN m${}^{-1}$ was obtained for Field’s metal, and 444 mN m${}^{-1}$ for EGaIn, the latter in close agreement with the values mentioned in the literature [15,21].

In the presence of O_2_, the pendant drops of Field’s metal were measured over a wide range of sizes, revealing different surface tension value, as previously reported [21,22]. This was attributed to the properties of the oxide skin, as yield stress causes the droplets to deviate from the behaviour of purely liquid droplets. As a result, a modification of the Laplace equation of equilibrium for a pendant drop was proposed by Xu et al. [21]:

(1) $$\sigma =\sigma_{0}+\tau_{Y}\cdot d$$

Based on Equation (1), an effective surface tension can be defined (σ). This can be split into two terms: the first one constant and associated to pure liquid surface tension ($\sigma_{0}$), and the second a function of the yield stress of the oxide skin ($\tau_{Y}$) and the characteristic length of the drop, usually defined by its diameter (*d*). In our experiments, similar results were obtained for surface tension with respect to *d*, including the linearity between such variables, but, intriguingly, we obtained values that were higher and also lower than $\sigma_{0}$ (417 mN m${}^{-1}$).

**Figure 15:**
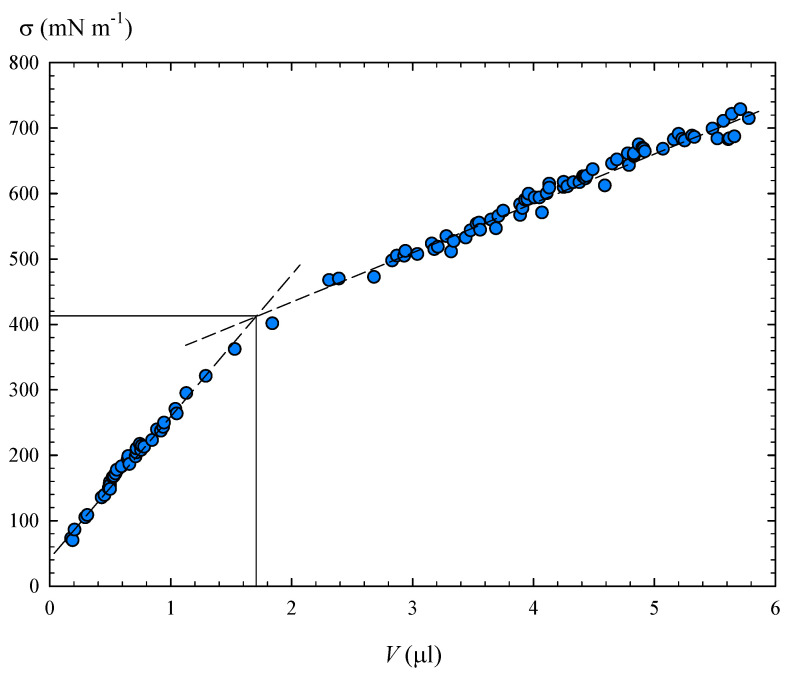
Apparent surface tension of Field’s metal as a function of drop volume.

Based on rheological results and previous studies, the droplets of these liquid metals behave as a viscoelastic membrane (oxide skin) coating on a Newtonian fluid (bulk). Thus, the surface properties of a membrane depend on the interior volume, and it is possible to define a critical volume that delimits the transition between elastic and viscoplastic behaviour [54]. That is why, for these liquid metals, the effective surface tension must also be related to the drop volume (V). For this reason, the surface tension data obtained with pendant drop tensiometry were analysed as a function of the drop volume; see Figure 15.

Figure 15 shows two different trends in the apparent surface tension and it can be observed how, around a certain volume, a transition occurs in the surface properties of Field’s metal. This also explains the difficulty in finding experimental values close to $\sigma_{0}$. Linear regressions (dashed lines) of the two trends were obtained (Figure 15). The cut-off point of the two regressions yielded a critical volume $V^{*}$ = 1.7 μL with an associated interfacial value of 412 mN m${}^{-1}$, which is quite similar to the $\sigma_{0}$ value obtained for the liquid metal without oxide skin (Figure 14).

**Figure 16:**
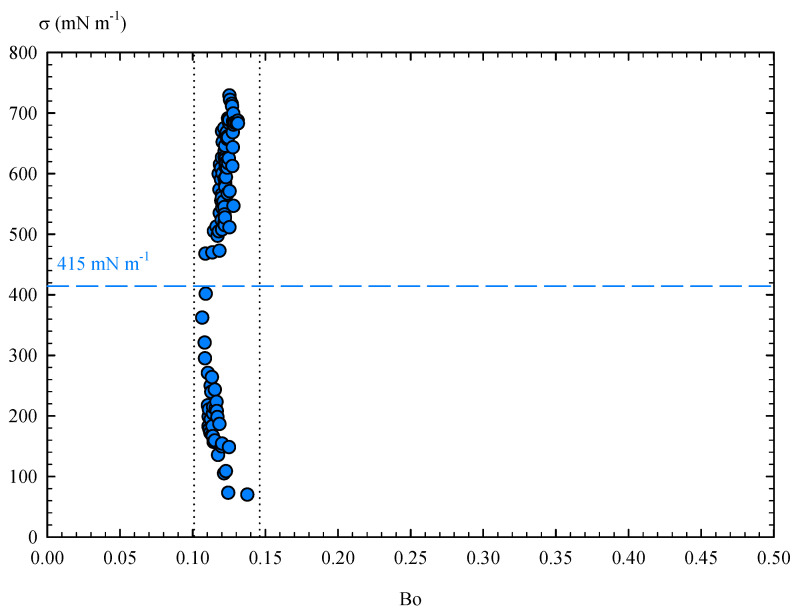
Surface tension of Field’s metal as function of the Bond number (Bo).

However, it should also be noted that the accuracy of ADSA algorithms may decrease when dealing with low Bond numbers [38,43]. In the pendant drop experiments with Field’s metal, we obtained Bond numbers between 0.106 and 0.138. Surface tension data were represented according to the Bond number (Figure 16), where two opposite trends can be clearly observed. Indeed, there is a range of surface tensions where Bo takes minimum values, statistically providing an average critical surface tension value of 413 mN m${}^{-1}$. This value again corresponds to $\sigma_{0}$, which can finally be established at 415 ± 3 mN m${}^{-1}$. As a result, the surface tension data measured for Field’s metal may vary due to its low number of Bond, although the viscoelastic contribution of the oxide layer seems to have a stronger influence on the results. Therefore, for drop sizes below a certain critical value, elastic effects can result in apparent surface tension values lower than $\sigma_{0}$, while, with larger drop sizes, the oxide skin exceeds its yield stress and acquires permanent deformations, also leading to apparent values of σ that are higher than $\sigma_{0}$.

## 4. Conclusions

This work describes an experimental study that was carried out to determine the rheological and surface properties of Field’s metal, with the aim of evaluating the suitability of this liquid metal for future applications involving microdroplet deposition. In addition, these properties are compared to those of EGaIn.

- Regarding the rheological properties, the results point to the lower viscosity of Field’s metal compared with EGaIn in the liquid state, and also to the elasticity of the oxide skin, which predominates over the viscoelastic behaviour in both cases.
- Torsional flow tests allowed us to determine the yield stress ($\tau_{Y}$) of the oxide skin—an essential process parameter—as well as the critical shear rate needed to start the shear flow of these materials. Concerning the evolution of the yield stress with regard to the shear rate, it is concluded that Field’s metal requires a lower shear stress to flow, although its shear flow is somewhat less stable than that of EGaIn. In addition, by means of torsional flow tests with peak hold, it was possible to analyse the time needed to reach the maximum steady shear stress.
- The surface properties of Field’s metal were also studied in order to evaluate the capacity to generate microdroplets, especially in “Drop on Demand” processes. Therefore, the solidification angle of Field’s metal on different substrates was analysed at different temperatures and, both alone and in the presence of the oxide skin. It was concluded that the oxide layer reduces the solidification angle of Field’s metal, with maximum reductions of approximately 8°.
- The surface tension of both liquid metals was analysed using the drop pendant method. In the absence of oxides, a well-defined and fixed surface tension value was obtained for the bulk of the material. By contrast, when these metals are oxidized, the viscoelastic properties of the oxide layer affect the interfacial stress values, producing apparent values that may be lower or higher than those corresponding to the bulk of the material. Thus, for small droplet volumes, surface tension values lower than that of the bulk were obtained, as the elasticity of the oxide skin contributes to the formation of drop shapes with a surface energy lower than that expected for a pure liquid. By contrast, above a certain drop volume, the surface tension overcomes that of the bulk, since the yield stress of the oxide layer is exceeded, and permanent deformations appear. Such deviations in the surface properties were identified by analysing the surface tension results of pendant drop experiments as a function of droplet volume and the Bond number.
- In the rheological study, the flow temperature ramp test demonstrated the potential usefulness of Field’s metal as an appropriate substitute for Ga alloys at temperatures above 338 K, since this metal remains rheologically stable up to temperatures close to 438 K. This would make Field’s metal suitable for “Drop on Demand” applications at high temperatures, as well as for combined use with liquid alloys, as already suggested by Shaikh et al. [55].

## Figures and Tables

**Table 1:** Test parameters of the rheological experiments.

Oscillatory Sweep	
Amplitude sweep	Frequency: 1, 10 Hz
	Strain range: from $10^{-3}$ to $10^{1}$% strain
**Torsional Flow**	
Peak hold	Time: 60 s
	Shear rate peaks: $10^{-3}$, $5\times 10^{-3}$, $10^{-2}$, $5\times 10^{-2}$ and $5\times 10^{-1}$ s${}^{-1}$
Steady flow	Sampling period: 5 s (20 points per decade)
	Shear rate range: from 10${}^{-4}$ to 10${}^{2}$ s${}^{-1}$
Flow ramp	Duration: 500 s
	Shear rate range: from $5\times 10^{-4}$ to $5\times 10^{1}$ s${}^{-1}$

**Table 2:** Binding energy values (eV) corresponding to the fitted curve peaks presented in Figure 2 (reference values along with the corresponding bibliographical sources are included for each component in parentheses).

	Bi 4f7 (eV)		In 3d5 (eV)		Sn 3d5 (eV)
Bi (156.93 [46])	156.84	In (444.00 [47])	443.97	Sn (485.00 [48])	485.00
Bi_2_O_3_ (159.80 [49])	159.81	In_2_O_3_ (444.90 [50])	445.06	SnO (486.00 [51])	485.99
				SnO_2_ (487.30 [52])	487.15

**Table 3:** Thermal analysis of the Field’s metal.

Field’s Metal	Specific Heat (J kg${}^{-1}$ K${}^{-1}$)	Melting Point (K)	Latent Heat (kJ kg${}^{-1}$)	
Solid	287.0	333.71	27.115	
Liquid	217.6	333.71	-	

**Table 4:** Rheological parameters obtained in this study and previous works.

	Field’s Metal	EGaIn		
	Experiments	Experiments	Dickey [15]	Xu [21]
$G^{\prime }$ (Pa)	5358 ± 147	5548 ± 141	5000 … 6000	-
$G^{\prime \prime }$ (Pa)	84 ± 6	98 ± 14	-	-
$G_{S}^{\prime }$ (N m${}^{-1}$)	16.07 ± 0.44	16.64 ± 0.42	13.3 ± 2.6	-
$G_{S}^{\prime \prime }$ (N m${}^{-1}$)	0.25 ± 0.02	0.29 ± 0.04	-	-
$\tau_{Y}$	57 ± 5	87 ± 5	-	108 ± 9

**Table 5:** Measurements of the contact angle *θ_c_* and the solidification angle *θ_s_* of Field’s metal and EGaIn over different substrates, in presence of O_2_ and N_2_.

		Field’s Metal				EGaIn		
		Solidification Angle *θ_s_*		Contact Angle *θ_c_* (358 K)		Contact Angle *θ_c_* (298 K)		
Material	*R_a_* (μm)	O_2_	N_2_	O_2_	N_2_	O_2_	N_2_	O_2_ Boley [30]
Glass	0.017	130.5 ± 0.9	136.4 ± 1.8	132 ± 0.5	138.8 ± 1.9	128.5 ± 1.8	139.4 ± 1.4	128.6 ± 1.3
AISI 316L	0.032	131.2 ± 1.4	139.3 ± 0.7	133.5 ± 0.7	141.1 ± 0.9	133.8 ± 0.1	137.9 ± 0.4	-
PTFE	0.431	137.5 ± 0.2	139.2 ± 0.3	139.3 ± 0.7	140.1 ± 0.3	132.2 ± 1.2	134.5 ± 1.2	-
Resin	4.864	138 ± 1.6	141 ± 0.3	141.1 ± 0.7	143.4 ± 0.1	152.1 ± 0.8	156 ± 0.2	-

## Data Availability

Not applicable.

## Nomenclature

Bo	Bond number
*d*	Maximum drop diameter (mm)
$G^{\prime }$	Elastic modulus (Pa)
$G_{S}^{\prime }$	Surface elastic modulus (Nm${}^{-1}$)
$G^{\prime \prime }$	Viscous Modulus (Pa)
$G_{S}^{\prime \prime }$	Surface viscous modulus (Nm${}^{-1}$)
*H*	Gap height (mm)
*t*	Time (s)
*T*	Substrate temperature (K)
*V*	Drop volume (μL)
$V^{*}$	Critical drop volume (μL)
γ	Shear strain (%)
δ	Phase angle
η	Shear viscosity (Pa· s)
$\theta_{c}$	Contact angle
$\theta_{s}$	Solidification angle
ρ	Density (kg m${}^{-3}$)
σ	Surface tension (mN m${}^{-1}$)
$\sigma_{0}$	Pure liquid surface tension (mN m${}^{-1}$)
$\tau_{Y}$	Yield stress (Pa)
